# Pyometrocolpos in an 18-Month-Old Child: A Rare Cause of Acute Abdomen

**DOI:** 10.7759/cureus.25710

**Published:** 2022-06-07

**Authors:** Avir Sarkar, Maninder K Ghotra, Isha Wadhawan

**Affiliations:** 1 Obstetrics and Gynaecology, All India Institute of Medical Sciences New Delhi, New Delhi, IND; 2 Obstetrics and Gynaecology, Fortis Escorts Hospital, Faridabad, IND

**Keywords:** acute abdomen, mullerian abnormality, hymenotomy, microperforate hymen, pyocolpos

## Abstract

Acute abdomen secondary to pyometrocolpos as a result of microperforate hymen in an 18-month-old child is extremely rare to witness. Such a child was admitted with a history of poor appetite, lethargy, high-grade febrile episodes, frequent urination, and multiple episodes of vomiting in seven days. There was no relief of symptoms on oral antibiotics. On careful examination, the abdomen was distended. A suprapubic bulge with mild tenderness was palpable. Genital examination revealed the absence of vaginal introitus with a bulging membrane. Ultrasound showed the presence of echogenic contents within the dilated uterine and vaginal cavities. An emergency hymenotomy drained 150 cc of malodorous purulent material. Symptoms were relieved post-surgery. This, to our knowledge, being the youngest case of microperforate hymen presenting with acute abdomen makes it worth reporting.

## Introduction

Imperforate hymen is a rarely occurring obstructive congenital anomaly of the genital tract. This clinical entity is usually diagnosed near menarche, usually presenting as amenorrhea and cyclical abdominal pain. It is usually associated with other developmental abnormalities of the urogenital system. Our case is an uncommon presentation of obstructive abnormality of the genital system at 18 months of life. Although imperforate hymen is not a rare occurrence in young girls, its presentation in early childhood with pyometrocolpos leading to acute abdomen is extremely rare in literature [1]. Oftentimes, physicians neglect to perform thorough genital examinations of newborns [2]. If imperforate hymen remains undiagnosed for a long time, it can become infected resulting in pyocolpos. As a result, such acute emergencies are liable to arise in childhood with undiagnosed imperforate hymen. This case clearly indicates the necessity for conducting a detailed genital examination of all neonates at the time of birth.

## Case presentation

An 18-month-old child was admitted to the emergency department with complaints of poor appetite, lethargy, high-grade febrile episodes, frequent urination, and multiple episodes of vomiting for seven days. There were no symptoms and signs of upper respiratory tract infection or meningitis. Being suspected of a case of uncomplicated urinary tract infection (UTI), she was earlier treated with antibiotics as an outpatient by a local pediatrician. With no relief of symptoms on oral antibiotics, her parents sought gynecologic consultation. 

On physical examination, she was febrile (101^0 ^F), with tachycardia of 150 beats/minute. The respiratory rate was normal, and the chest was bilaterally clear. The abdomen was mildly distended with a suprapubic bulge and rebound tenderness. Genital examination revealed an absence of vaginal introitus with a bulging membrane. Urethral and anal orifices were normally situated. Thereby, differential diagnoses of imperforate and microperforate hymen were considered.

**Figure 1:**
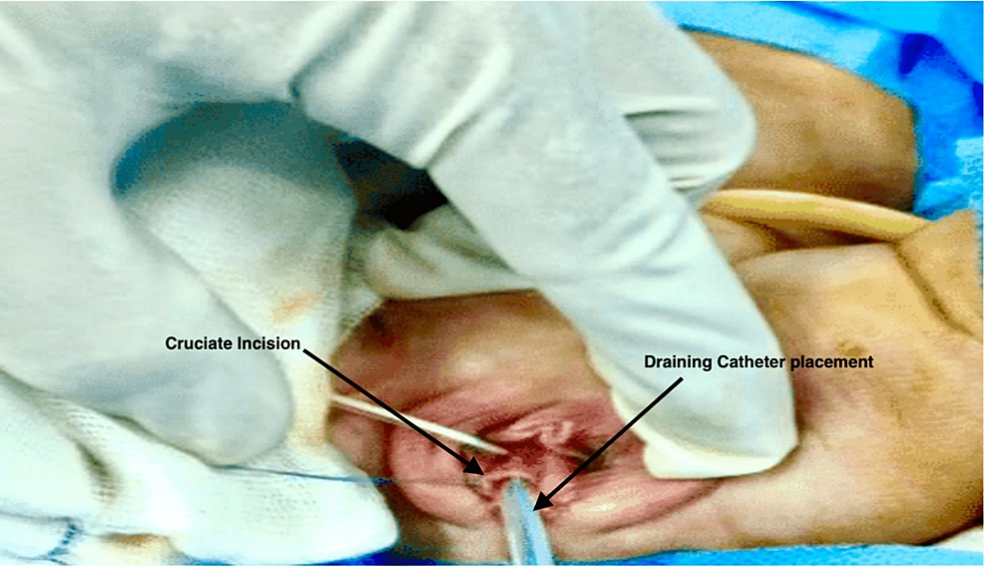
Cruciate incision over the hymen in the 18-month-old with a placement draining catheter.

Pelvic ultrasound revealed a dilated vagina and endometrial cavity with echogenic contents. Both adnexa and kidneys were normal. A routine and microscopic examination of a clean catch urine sample were within normal limits with less than five leukocytes per high power field. The urine culture was sterile. The total leucocyte count was 19,000/mm^3^. Other laboratory investigations were unremarkable. Empirical treatment with intravenous antibiotics (injections of cefotaxime and Metrogyl) was initiated. A magnetic resonance imaging with three-dimensional resolution showed a grossly expanded fluid-filled vaginal cavity measuring 9.0*4.3*4.5 cm. The endometrial cavity was also distended to 3.5 cm. Fluid was hypointense in T1 and hyperintense in T2 sequences. A hymenotomy was performed through a cruciate incision, and around 150 mL of malodorous purulent material was drained (Figure 1).

Inoculum from pus culture showed a growth of *E. coli*, antibiotics were changed to injection piperacillin and tazobactam. The fever subsided in the postoperative period and the patient was discharged on the eighth day in stable condition. She is doing well and is showing age-appropriate growth at follow-up visits. Repeat ultrasounds at two months and 24-months post-hymenotomy were unremarkable. 

## Discussion

Pyometrocolpos may arise secondary to imperforate hymen or transverse vaginal septum, which may be related to the failure of apoptosis of the genital tract due to abnormal genetic predisposition or an altered hormonal milieu [3]. Maternal estrogen accelerates the production of cervicovaginal secretion which acts as a fertile medium for bacterial colonization. Symptoms like dull aching pain abdomen, fever, and poor growth can develop insidiously unless there is compression upon the ureters causing bilateral hydronephrosis or the rectum causing bouts of intestinal obstruction [1]. Failure to perform a thorough genital examination in a newborn can lead to the development of such acute emergencies. In our case, massive pyometrocolpos causing colonic and rectal compression was the probable cause of the symptoms and signs of acute abdomen. 

The treatment strategy includes drainage of collected purulent material which is usually done by hymenotomy [4]. With ultrasonography, diagnosis of such obstructive Müllerian anomalies is quite easy. Prenatal diagnosis in a 25-week-old fetus has also been reported [5]. Although the diagnosis of an imperforate hymen is not so rare with a reported incidence ranging from 0.0014 to 0.1% [6], this case constitutes a novelty in itself. To the best of our knowledge, this is the youngest case of obstructive Müllerian anomaly presenting with acute abdomen (at 18 months of age) [7]. The rarity of its presentation makes the case worth reporting. 

## Conclusions

Obstructive Müllerian anomaly is not a very common entity and is often missed due to a lack of thorough genital examination at birth. Due to the rarity of cases, routine screening at birth is not recommended. Late consequences can cause acute urinary and intestinal obstructions. Since the diagnosis and treatment are quite easy, postsurgical prognosis is fairly good. Moreover, emphasis should be laid on accurately diagnosing imperforate and microperforate hymen and not misdiagnosing it as a vaginal septum or agenesis since the mode of management is completely different.
